# Socio-demographic predictors of obesity among 12,975 adult ever married Egyptian women of reproductive age group: evidence from nationwide survey

**DOI:** 10.1186/s12889-023-17397-7

**Published:** 2023-12-13

**Authors:** Amr Ehab El-Qushayri, Md Anwar Hossain, Imteaz Mahmud, Mohammad Rashidul Hashan, Rajat Das Gupta

**Affiliations:** 1 Faculty of Medicine, Minia University, Minia, 61519 Egypt; 2 Gladstone Hospital, QLD- 4680, Gladstone, Australia; 3 Department of Public Health, North South University, Dhaka, 1229 Bangladesh; 4 Directorate General of Health Services, Ministry of Health and Family Welfare, Government of Bangladesh, Dhaka, 1212 Bangladesh; 5 School of Medical, Health and Applied Sciences, Central Queensland University, Queensland, Australia; 6 BRAC James P Grant School of Public Health, BRAC University, 6th Floor, Medona Tower, 28 Mohakhali Commercial Area, Bir Uttom A K Khandakar Road, Dhaka, 1213 Bangladesh; 7 Department of Epidemiology and Biostatistics, Arnold School of Public Health, University of South Carolina, Columbia, SC United States of America

**Keywords:** Obesity, Women, Egypt, DHS

## Abstract

**Aim:**

We aimed to explore the predictors associated with obesity among adult ever-married Egyptian women aged 20–49 years based on the Egyptian Demographic and Health Survey (EDHS).

**Method:**

We included adult ever married women from the EDHS conducted in 2014 that initially recruited 21,903 women. Univariate and multivariable analysis was conducted to identify socio-demographic predictors of women’s obesity.

**Result:**

We included 12,975 Egyptian women. Among them, 76% of the total respondents were obese where as 24% were with normal body mass index (BMI). In multivariable analysis, the results revealed that increasing age, higher wealth index, listening to radio at least once a week and women with primary and secondary education were at significant odds of developing obesity (*p* < 0.05). However, we found no association between residence of participants and the frequency of watching television upon the development of obesity (*p* > 0.05).

**Conclusion:**

Appropriate and targeted interventions should be implemented among the Egyptian reproductive age women to reduce the obesity as well as non-communicable diseases load associated with obesity. National Health Service policy makers should take multilevel approach targeting high risk sub-groups to raise awareness and to provide prevention against obesity and the subsequent complications.

## Introduction

Worldwide, approximately 2 billion adults are overweight, with 677.6 million obese among them [1]. Among the obese population, the majority of them are women (284.1 million are men and 393.5 million are women) [2]. It has a number of long-term complications, like cardiovascular disease (hypertension, heart disease, and stroke), type 2 diabetes, cancers, and other non-communicable diseases (NCDs) [3–5], subsequently resulting in premature mortality, loss of productivity, financial burden (on households, health systems, and entire economies), depression, and low self-esteem later in life [6, 7]. With the Covid-19 pandemic, obesity is found to be associated with higher risk of hospitalization and death of affected individuals. A recent meta-analysis including 75 international studies showed that obese adults had a 113% higher risk of hospitalization, a 74% higher risk of intensive care unit (ICU) admission and an additional 48% risk of death than their normal-weight counterparts [8]. The obesity crisis is putting a strain on the health-care system and have high economic burden. Obesity is estimated to cost the world economy about 2 trillion USD per year [9].

In general, women have a higher prevalence of overweight and obesity than men [10–12]. Overweight and obesity have been associated with different types of malignancies in women, including post-menopausal breast cancer, ovarian cancer, and endometrial cancer [13]. Obesity in mothers is linked to obesity in offsprings, resulting in an intergenerational effect on future generations [14]. Previously, overweight and obesity were considered to be an issue only in high-income nations. However, over the time, it has become a global problem affecting even low-and middle-income countries [3]. Overweight and obesity are serious problems in various parts of the world, particularly in the Eastern Mediterranean regions (EMR). As a result, NCDs account for half of all fatalities in this region [14, 15]. Egypt is a country in the EMR area with an alarming rate of obesity among adults. A study published in the New England Journal in 2017 conducted on 195 countries and including children and adults, showed that more than a third (35.3%) of the Egyptian adults were obese, which is the highest among all nations [16]. In Egypt, females have a considerably greater prevalence of obesity than males (41.1% compared to 22.7%) [17]. More notably, this rate has increased at an average annual growth rate of 1.89% over the past 20 years [17]. Obesity is responsible for the high rate of different NCDs, which are estimated to account for 82% of all fatalities and 67% of premature deaths in Egypt [17]. With an aging and increasing population, as well as the burden of obesity and other NCDs, Egypt faces a significant problem in its health-care system and financial loss [18]. A systematic review aimed to identify possible reasons for obesity in the EMR regions found that nutrition transition, inactivity, urbanization, marital status, a shorter duration of breastfeeding, frequent snacking, skipping breakfast, a high intake of sugary beverages, an increase in the incidence of eating outside the home, long periods spent watching television, massive marketing promotion of high-fat foods, stunting, perceived body image, cultural elements, and food subsidized policy were associated with an increased risk of obesity [19]. Another study, based upon Egyptian Demographic and Health Surveys (EDHS) from 1992 to 2008, reported an association between education and household wealth status with obesity over time [20].

However, there is a lack of recent evidence on the socioeconomic, behavioural, and reproductive factors associated with obesity. In this analysis, we aimed to explore the socio-demographic correlates of obesity among adult ever-married Egyptian women.

## Method

### Data source

We conducted a population based study by obtaining the data from the standard (EDHS) questionnaire conducted in Egypt during 2014. The survey initially collected 21,903 eligible women aged 15–49 years old for participation across the 25 governments. Many anthropometric measures were collected from the included women in particular body mass index (BMI).

#### Inclusion criteria

We included adult ever married women aged 20–49 years old that had the required information regarding BMI measured by kg/m^2^ per the guideline of the Centers for Disease Control And Prevention (CDC) [21, 22].

#### Exclusion criteria

We excluded ever married women who had missing data regarding BMI, underweight women (BMI < 18.5), overweight women (BMI 25–30) and women aged below 20 years old.

### Included variables

Many socio-demographic variables were extracted from the survey including: Age (divided into five years groups), residence (rural or urban), wealth index profile that was categorized by the DHS board into (richest, richer, middle, poorer and poorest), educational level (higher education, secondary education, primary education and no education) and frequency of listening to radio or watching television (at least one a week, less than once a week and not at all).

### Statistical analysis

SPSS version 24 was used for the data analyses. As regard the nature of the included variables, we used the chi-square test for identifying the difference between the obese group and the normal population group after weighting the data based upon the DHS recommendations and the data was reported as frequency and percentages. Moreover, to identify the predictors of women obesity, we conducted logistic regression analysis including univariate and multivariable analysis (with control of all potential covariates) and the results were reported as odds ratio (OR) and 95% confidence interval (95%CI). Furthermore, we set a cut off value of five regarding the variance inflation factor for testing the multicollinearity between all the independent variables. We considered statistical significant results when *p* value falls below 0.05.

## Results

For the analysis, a set of data was collected from 12,975 Egyptian reproductive aged (20–49 years) women. Among them, 76% of the total respondents were obese whereas 24% women were with normal BMI (Table 1).

**Table 1:** Characteristics of the included women according to BMI

Variables		BMI		*P* value
		Normal (BMI = 18.5: 25) N = 3111	Obese (BMI > 30) N = 9864	
**Age in 5 year groups (%)**	**20–24**	935 (30.1)	744 (7.5)	**< 0.001**
	**25–29**	936 (30.1)	1697 (17.2)	
	**30–34**	546 (17.6)	1905 (19.3)	
	**35–39**	327 (10.5)	1921 (19.5)	
	**40–44**	193 (6.2)	1822 (18.5)	
	**45–49**	172 (5.6)	1773 (18)	
**Highest educational level (%)**	**No**	750 (24.1)	2620 (26.6)	**0.001**
	**Primary**	273 (8.8)	1098 (11.1)	
	**Secondary**	1662 (53.4)	4893 (49.6)	
	**Higher**	425 (13.7)	1252 (13.7)	
**Type of place of residence (%)**	**Urban**	879 (28.3)	3616 (36.7)	**< 0.001**
	**Rural**	2232 (71.7)	6248 (63.3)	
**Wealth index (%)**	**Poorest**	700 (22.5)	1615 (16.4)	**< 0.001**
	**Poorer**	667 (21.5)	1923 (19.5)	
	**Middle**	730 (23.5)	2187 (22.2)	
	**Richer**	521 (16.8)	2215 (23.5)	
	**Richest**	492 (15.8)	1922 (19.5)	
**Frequency of listening to radio (%)**	**Not at all**	2497 (80.3)	7426 (75.3)	**< 0.001**
	**Less than once a week**	134 (4.3)	548 (5.6)	
	**At least once a week**	477 (15.4)	1887 (19.1)	
**Frequency of watching television (%)**	**Not at all**	62 (2)	152 (1.5)	0.14
	**Less than once a week**	71 (2.3)	180 (1.8)	
	**At least once a week**	2975 (95.7)	9531 (96.6)	

The result showed that the highest proportion of obese participants was from 35 to 49 years age group which contained 56% of total obese women. However, 60.2% of normal BMI females’ age was in the range of 20–29 years old.

Among the women with normal BMI, more than half completed secondary education and 8.8% and 13.7% completed primary and higher education, respectively. About a quarter had no education in the entire sample. Considering the obese women, half of the respondents earned the secondary level education, where the participant with primary and higher education were 11.1% and 13.7% respectively.

Considering residence, nearly three quadrants (71.7%) of normal weight women resided in rural households compared to 63.3% of obese women. Obesity was more predominant among the richer wealth quintile (23.5%) group followed by middle, richest, poorer and poorest (22.2%, 19.5%, 19.5% and 16.4% respectively). On the other hand, the highest proportion of women with normal BMI was from middle group (23.5%).

We found that nearly four fifths of the study participants did not listen to radio at all, whereas only 4.3% and 5.6% of participants listened to radio less than once a week and 15.4% and 19.1% listened to radio least once a week among normal weighted and obese women, respectively. Our results also demonstrated that the majority of the women with normal BMI (95.7%) and obesity (96.6%) watched television at least once in a week which showed no significance in the analysis.

In multivariable analysis (Table 2), the odds of obesity was increased with increasing age of the participants, 25–29 years (OR: 2.41; 95%CI: 2.12–2.57; *p* < 0.001), 30–34 years (OR: 5.06; 95%CI: 4.40–5.83; *p* < 0.001), 35–39 years (OR: 8.80; 95%CI: 7.50–10.3; *p* < 0.001), 40–44 years (OR: 14; 95%CI: 11.7–16.8; *p* < 0.001), 45–49 years (OR: 15.6; 95%CI: 12.9–18.8; *p* < 0.001). Women with primary and secondary but not higher education had higher odds of developing obesity (OR: 1.23; 95%CI: 1.04–1.46; *p* = 0.016), (OR: 1.23; 95%CI: 1.09–1.39; *p* = 0.001), (OR: 0.95; 95%CI: 0.80–1.12; *p* = 0.5), respectively. Along with the improvement of wealth quintiles the group of participants who belong to poorer (OR: 1.33; 95%CI: 1.16–1.53; *p* < 0.001), middle (OR: 1.64; 95%CI: 1.42–1.89; *p* < 0.001), richer (OR: 2.08; 95%CI: 1.76–2.46; *p* < 0.001) and richest households (OR: 1.76; 95%CI: 1.44–2.16; *p* < 0.001) had a higher possibility of developing obesity relative to the poorest wealth quantile group of women. In terms of residence, we did not find an association between rural residence and the increased odds of obesity (OR: 1.04; 95%CI: 0.90–1.20; *p* = 0.61). The respondents of the study who listened to radio at least once a week (OR: 1.30; 95%CI: 1.17–1.47; *p* < 0.001) were found more likely to be obese comparing with those who never listen to radio. We also found that watching television was not significantly associated with obesity (*p* > 0.05).

**Table 2:** Predictors of women obesity

Variables		Univariate		Multivariable	
		OR (95% CI)	*P* value	OR (95% CI)	*P* value
**Age in 5 year groups**	**20–24**	**Reference**			
	**25–29**	2.37 (2.08–2.70)	**< 0.001**	2.41 (2.12–2.57)	**< 0.001**
	**30–34**	4.78 (4.17–5.49)	**< 0.001**	5.06 (4.40–5.83)	**< 0.001**
	**35–39**	8.20 (7.04–9.57)	**< 0.001**	8.80 (7.50–10.3)	**< 0.001**
	**40–44**	12.8 (10.8–15.3)	**< 0.001**	14.0 (11.7–16.8)	**< 0.001**
	**45–49**	13.92 (11.6–16.7)	**< 0.001**	15.6 (12.9–18.8)	**< 0.001**
**Highest educational level**	**No**	**Reference**			
	**Primary**	1.13 (0.97–1.32)	0.12	1.23 (1.04–1.46)	**0.016**
	**Secondary**	0.84 (0.76–0.93)	**0.001**	1.23 (1.09–1.39)	**0.001**
	**Higher**	0.81 (0.70–0.92)	**0.002**	0.95 (0.80–1.12)	0.5
**Type of place of residence**	**Urban**	**Reference**			
	**Rural**	0.66 (0.61–0.71)	**< 0.001**	1.04 (0.90–1.20)	0.61
**Wealth index**	**Poorest**	**Reference**			
	**Poorer**	1.20 (1.06–1.36)	**0.004**	1.33 (1.16–1.53)	**< 0.001**
	**Middle**	1.25 (1.11–1.42)	**< 0.001**	1.64 (1.42–1.89)	**< 0.001**
	**Richer**	1.75 (1.54–1.99)	**< 0.001**	2.08 (1.76–2.46)	**< 0.001**
	**Richest**	1.74 (1.54–1.97)	**< 0.001**	1.76 (1.44–2.16)	**< 0.001**
**Frequency of listening to radio**	**Not at all**	**Reference**			
	**Less than once a week**	1.23 (1.02–1.47)	**0.033**	1.06 (0.86–1.29)	0.69
	**At least once a week**	1.46 (1.30–1.64)	**< 0.001**	1.30 (1.14–1.47)	**< 0.001**
**Frequency of watching television**	**Not at all**	**Reference**			
	**Less than once a week**	1.02 (0.68–1.55)	0.9	0.99 (0.63–1.57)	0.9
	**At least once a week**	1.28 (0.93–1.76)	0.13	1.25 (0.88–1.76)	0.21

## Discussion

To the best of our knowledge, this study is the first to detect the factors of obesity among adult women in Egypt by using a nationally representative data.

In this study, the analysis revealed a significant association between age and obesity. We found that the prevalence of obesity was higher among older age group for instance; the odds were 15 times higher among 44–49 years old women than the women aged 20–24 years. The study finding is consistent with other studies using the data of many countries, where a higher prevalence of obesity was found in older age group of women [23–25]. Less physical activity and high-energy food intake might be the probable cause of obesity among older age women [26]. Along with the advancement of age the body composition may change for instance the fat composition may increase [27, 28]. Therefore, proper interventions are essential to prevent obesity by targeting the modifiable risk factors.

The analysis of the study revealed a positive association between educational level and obesity. After adjusting other factors, both primary and secondary educated women were at higher odds of being obese in compare to illiterate group. Women with higher education were found to be at the lowest odds among all. Evidence from other global study showed the similar findings of better health condition among higher educational group because of their high socioeconomic status, health awareness, self-control and better knowledge on nutrition [29–32]. Finding of our study was different than some other studies (e.g. Ghana, Bangladesh, Ethiopia), where reproductive-age women with secondary or higher education were found more likely being overweight or obese [33–35].

An important factor in this study was the place of residence. We found that rural women had higher odds of developing obesity; however the comparison did not yield statistical significance. The result of the study is contrary to some other previously published studies in lower middle income countries (LMICs) and developing countries [31, 36–38]. Urban life tends to be cost therefore, the participation of women to the household’s income is pivotal at which the women prefer work engagement which requires more physical activity [39]. Moreover, urban women favoured employment to avoid the violence that was expressed from the husband/partner [40]. However, the family income in the rural communities is low in Egypt, that prevents them from eating high quality healthy food and shifting to ingest high carbohydrate diet which increase the susceptibility to obesity [41].

In our study we found that the women with high wealth quintile were more presumably to be obese than the women who belonged poor households. Similar findings were consistent with other studies from different low and middle income countries where wealth was a significant indicator of obesity [25, 35, 42, 43]. In some African and other countries, people have a perception that larger body size or obesity is a symbol of wealth, leading them to consume enough foods and high-calorie diets [37, 42, 44]. On the other hand, some studies conducted in high income countries revealed that the wealthy women are less likely to develop obesity [45, 46]. Our study findings recommend the intervention for the affluent obese population to lessen the obesity prevalence and its complications.

The analysis of our study indicated that the odds of being obese is more among the women who listen to radio than the women who did not listen to radio at all. Individuals, who listen to radio may not involve in enough physical activity to keep them fit and this habit may habituated them with sedentary life style which proved the associated between radio listening and obesity [47]. Our study suggest that the appropriate action need to be taken using traditional and social media focusing the obese Egyptian women to raise the awareness about the bad effect of less physical activities and listening to radio.

Significant association between television watching and obesity was not found in our study result. However, some previous studies performed with the data of Ghana, Bangladesh, Myanmar, India, and Nepal illustrated significant association between obesity and television watching [48–51]. The reason behind this association is that the women who spend more time watching television usually do less physical activity, sit for long time and take unhealthy food television [49, 50, 52].

The strongest point of our study is that, we used nationally representative data to detect the predictors of obesity among the Egyptian reproductive aged women. Furthermore, as the Egyptian demography and health survey data collection tool is standard and validated, so, the possibility of biasness and error was less than other small-sized studies.

We encountered several limitations that need future assessment by further studies. At first, the design of this study was cross-sectional in nature therefore we cannot determine the true association between our outcome, it is risk factors or complications. Secondly, The survey was only limited to participants up to 49 years old, which increased the need for future studies for including more elderly participants. Thirdly, the survey data does not contain the data of comorbidity that can play a crucial role in obesity development. Fourthly, only ever married women were only eligible in our study, therefore we could not add marital status as one of the predictors of obesity.

### Recommendations

Globally, overweight and obesity are the sixth leading cause of Disability Adjusted Life Years (DALYs) [3]. Until now, Egypt does not have a nationwide program to reduce obesity burden. Despite that, some local services were offered to decrease such morbidity [53]. Some interventions such as regular screening, poverty reduction, improve nutrition knowledge and lifestyle modification approach can be beneficial. National Health Service policy makers should take multilevel approach to raise awareness, arrange campaign and seminar to improve the health condition and prevent obesity as well as associated NCDs.

## Conclusion

In conclusion, women from older age, low educational level, high wealth index, and frequent radio listening group had higher odds of being obese. Appropriate and targeted interventions should be implemented among the Egyptian reproductive age women to reduce the obesity as well as NCDs burden associated with obesity.

## Data Availability

Data is available upon request from the website of ICF International (https://dhsprogram.com/data/available-datasets.cfm).
